# *Buchholzia coriacea* seed extract attenuates mercury-induced cerebral and cerebellar oxidative neurotoxicity via NO signaling and suppression of oxidative stress, adenosine deaminase and acetylcholinesterase activities in rats

**DOI:** 10.22038/AJP.2021.18262

**Published:** 2022

**Authors:** Simeon I Egba, Ademola C Famurewa, Lilian E Omoruyi

**Affiliations:** 1 *Department of Biochemistry, Michael Okpara University of Agriculture, Umudike, Abia State, Nigeria*; 2 *Department of Medical Biochemistry, Faculty of Basic Medical Sciences, College of Medicine, Alex-Ekwueme Federal University, Ndufu-Alike, Ikwo, Ebonyi State, Nigeria*

**Keywords:** Buccholzia coriacea, Neurotoxicant, Mercury, Neurotoxicity, Oxidative stress

## Abstract

**Objective:**

Mercury (Hg) is a classic cumulative neurotoxicant implicated in neuronal deficit via oxidative damage and inflammatory responses. We sought to investigate whether *Buccholzia coriacea* seed methanol extract (BCSE) would modulate oxidative neurotoxicity induced by Hg in rats.

**Materials and Methods:**

Rats were orally treated with BCSE (200 or 400 mg/kg body weight of rat) for 28 days, while Hg was administered from day 15 to day 28. After sacrifice, antioxidant enzyme activities, reduced glutathione (GSH), nitric oxide (NO), malondialdehyde (MDA), and acetylcholinesterase (AchE) and adenine deaminase (ADA) activities were evaluated in the cerebrum and cerebellum of rats.

**Results:**

Mercury induced significant depressions in catalase (CAT) and glutathione peroxidase (GPx) activities and GSH levels, whereas levels of NO and activities of AchE and ADA markedly increased. The histopathology of the brain tissues confirmed these changes. In contrast, BCSE administration prominently modulated the brain NO production and reversed the Hg-induced biochemical alterations comparable to normal control.

**Conclusion:**

Methanol extract of *B. coriacea *seeds protects the cerebrum and cerebellum against Hg-induced brain damage via its antioxidant and NO modulatory actions.

## Introduction

Mercury (Hg) is a hazardous non-essential metallic element of worldwide concern since the industrial revolution. Hg is a heavy metal of nature and its exposure is ubiquitous due to a wide spectrum of industrial applications in barometers, thermometers and dental fillings (Goudarzi et al., 2017 ▶). Coal-burning emissions and industrial wastes are recognized as major anthropogenic sources of mercury (Cardenas et al., 2017 ▶). These sources have significantly increased Hg level in seawater by three folds; seafoods including fish are well known sources of Hg exposure (Thiagarajan et al., 2018 ▶). Mercury is a multiorgan environmental toxicant that bioaccumulates along the food chain. Hg exposure represents a significant health challenge in humans and animals because of its toxicity and contribution to pathologies in various tissues. The target of inorganic Hg accumulation is the brain (Cardenas et al., 2017 ▶). Literature has implicated Hg in the pathogenesis of neurological deficit and behavioural dysfunction (Cardenas et al., 2017 ▶). It is associated with alterations to neurotransmitters and DNA methylation (Bakulski et al., 2015 ▶; Castoldi et al., 2003 ▶). Recent reports show the potential of Hg to induce neurotoxicity and neuronal cell death (Owoeye and Gabriel, 2017 ▶; Moneim, 2015 ▶). 

However, the mechanism of Hg toxicity is still unclear, and the specific underlying mechanism of Hg neurotoxicity remains poorly elucidated. But current data suggest several mechanisms of which, oxidative stress is well reported to play a leading role in Hg-induced neurotoxicity (Moneim, 2015 ▶; Mieiro et al., 2011 ▶). Several systematic investigations report that the neurotoxic prowess of Hg is in its ability to mediate increased reactive oxygen species (ROS) production (Mesquita et al., 2016 ▶). Findings have shown that Hg has strong affinity for glutathione-based enzymes by reacting with their thiol (-SH) group (Mesquita et al., 2016 ▶; Rao and Purohit, 2011 ▶). This reaction depletes intracellular thiols resulting in reduced antioxidant activities of reduced glutathione, glutathione peroxidase and glutathione reductase, thus aggravating ROS generation and oxidative damage. 

Recently, literature is emerging on the potential of plant products to mitigate Hg neurotoxicity by protecting neurons against oxidative stress and restoring redox balance. *Buchholzia coriacea* Engler known as magic cola is used for a myriad of therapeutic purposes in African folklore medicine (Adisa et al., 2011 ▶). The seeds of *B. coriacea* plant are topically applied to the stomach to manage difficult childbirth (Olaiya and Omolekan, 2013 ▶). Its efficacy for treatment of diabetes mellitus has been reported (Adisa et al., 2011 ▶). Previous studies show that the seed contains alkaloids, cardiac glycosides, saponins, and flavone glycosides (Adisa et al., 2011 ▶; Ajaiyeoba et al., 2001 ▶). However, to our knowledge, till date, there is no study evaluating the effect of *B. coriacea* seed extract on Hg neurotoxicity. Therefore, this experimental study was designed to clarify the role of *B. coriacea* seed extract in Hg-induced oxidative stress-mediated cerebral and cerebellar neurotoxicity in rats. 

## Materials and Methods

**Chemicals**

Mercury (II) chloride (HgCl_2_) was purchased from JHD Fine Chemicals, China. Thiobarbituric acid and trichloroacetic acid were purchased from Merck. Assay kits for biochemical indices were purchased from Randox Laboratory Ltd., UK. All other chemicals and reagents were of analytical grade. Double-distilled water was used as the solvent. 

**Plant material**

Fresh seeds of *B. coriacea* plant were purchased from a local market, Orio-Ugba market in Umuahia, Abia State, Nigeria. The seeds were immediately rinsed to remove dirt, and cut into small pieces, shade dried and oven dried at 40^o^C. The dried parts were then pulverized using a manual grinder. 

**Extraction of** ***B. coriacea*** **seeds**

 The powdered seeds (2 kg) were macerated in absolute methanol with intermittent shaking for 72 hr. The extract was then filtered using Whatman No 1 filter papers. The filtrate was concentrated at 40^o^C to obtain a solid extract that was then weighed.

**Animals**

Male Wistar rats (150-200 g), 6-8 weeks old used in this study, were obtained from the Laboratory Animal Unit of the Faculty of Veterinary Medicine, University of Nigeria, Nsukka. Rats were used for the *in vivo* study that required several administration and handling for 28 days. They were kept in well-ventilated stainless steel cages under a 12 hr light/dark cycle. All the rats were fed with standard commercial pelleted feed (Vital feed®, Nigeria) and tap water *ad libitum* for 2 weeks before the experimental treatment. The Animal Care Ethics Committee of the Department of Biochemistry, Michael Okpara University of Agriculture, Umudike, approved the experimental design, and the protocol conformed to the guidelines of the National Institute of Health (1985). 

**Acute toxicity study**

Acute toxicity and lethality study of *B. coriacea *seed methanol extract (BCSE) was determined using Locke’s method (1983). Mice were used to determine acute toxicity according to Locke’s method (1983) which has been published widely. This method has two phases, phase 1 and 2 respectively, thus affording the opportunity for effective monitoring for any neurobehavioral toxicity. The first phase required nine mice which were divided into three groups of three mice; the first group was given the extract at 10 mg/kg body weight; the second group was given the extract at 100 mg/kg body weight; and the third group was given the extract at 1000 mg/kg body weight. In the second phase, nine mice were also used and divided into three groups of three mice and administered the extract at 1600, 2900 and 5000 mg/kg body weight, as in phase 1. 

**Experimental design**

In the present study, rats were randomly divided into 5 groups (n=5). The experimental design for the *in vivo* study after 2-week acclimatization period was as follows:

Group 1 (Normal control): rats received normal saline (2 ml/kg body weight) for 28 days.

Group 2 (Extract control): rats received extract (400 mg/kg body weight, orally) for 28 days.

Group 3 (Hg control): rats received HgCl_2_ (4 mg/kg body weight, orally) for the last 14 days (Lakshmana et al., 1993).

Group 4 (Extract I + Hg): rats received extract (200 mg/kg body weight) for 28 days + HgCl_2_ (4 mg/kg body weight) for the last 14 days (day 15 to day 28).

Group 5 (Extract II + Hg): rats received extract (400 mg/kg body weight) for 28 days + HgCl_2_ (4 mg/kg body weight) for the last 14 days (day 15 to day 28). The extract was administered 90 min before the HgCl_2_ intoxication. 

At the end of the treatment period (28 consecutive days), overnight fasted animals were decapitated under mild diethyl ether anesthesia and the cranium was gently opened to remove the cerebrum and cerebellum. They were washed with an ice-cold saline solution, dried and separately homogenized in Bouin’s fluid (1:5 w/v, pH 6.4) and centrifuged at 4000 g for 20 min. The homogenate supernatant obtained was used to determine the biochemical indices evaluated in this study. The cerebrum and cerebellar tissues were immediately fixed in 10% buffered formalin for histopathological examinations. 

**Biochemical analyses**

In the cerebrum and cerebellum, activity of catalase (CAT) was evaluated according to Aebi method (1983) ▶, glutathione peroxidase (GPx) by Flohe and Gunzler method (1984) ▶, while reduced glutathione (GSH) level was determined by Jollow et al (1974) ▶. Malondialdehyde was estimated by measuring thiobarbituric acid reactive substances (TBARS) by method of Ohkawa et al. (1979) ▶. Acetylcholinesterase (AchE) activity was estimated by the method of Ellman et al. (1961) ▶, activity of adenosine deaminase (ADA) was determined by Giusti method (1974) ▶, while nitric oxide (NO) was determined according to the method described by Sreejayam (1997) ▶. Total cholesterol was estimated according to instructions in the Randox kits.

**Histopathology**

Tissue samples of cerebrum and cerebellum were fixed in 10% formalin and dehydrated in ethanol and then embedded in paraffin blocks. The blocks were cut into 5 μm sections using a microtome, fixed on slides followed with hematoxylin and eosin (H&E) staining. The prepared slides were observed under light microscope.

**Statistical analysis**

All the data are presented as mean±standard deviation. One-way ANOVA followed by the least-significant difference (LSD) was used for making comparisons among the groups. A value of p<0.05 was considered statistically significant.

## Results

**Acute toxicity study**

Acute toxicity study test (Table 1) did not show any mortality, morbidity or other apparent signs of toxicity at the doses (10 to 5000 mg/kg) used within 4 hr of continuous observation and after 24 hr. No salivation, diarrhea, lethargy or unusual behaviors were observed. Food and water intake, body weight and respiration were normal. Having these results in mind, 1/25th and 1/12.5th of the maximum dose (5000 mg/kg) were adopted for the study which gave rise to 200 and 400 mg/kg doses of the extracts used in the treatment groups, respectively. 

**Effect of BSCE on cerebral oxidative stress indices**

Table 2 shows the effect of *B coriacea *seed methanol extract (BCSE) on antioxidant enzymes CAT and GPx as well as GSH and MDA in the cerebrum of rats treated with Hg. It was observed that Hg administration significantly increased CAT activity, whereas GPx activity was depressed significantly (p<0.05) compared to the normal control group. Furthermore, Hg markedly reduced cerebral level of GSH accompanied with significant increases in MDA level compared to the normal control group (p<0.05). Interestingly, sub-acute prophylactic treatment with BCSE modulated CAT activity comparable to normal control, and the GPx activity and GSH level were elevated markedly only at 400 mg/kg dose of BSCE (p<0.05).

**Table 1:** Acute toxicity study and observation at 4 and 24 hours

Observation	Group 1 (0.01 g/kg)	Group 2 (0.1 g/kg)	Group 3 (1 g/kg)	Group 4 (1.6 g/kg)	Group 5 (2.9 g/kg)	Group 6 (5 g/kg)
Mortality	N	N	N	N	N	N
Morbidity	N	N	N	N	N	N
Salivation	N	N	N	N	N	N
Diarrhea	N	N	N	N	N	N
Lethargy	N	N	N	N	N	N

N: None; n=3/group

**Table 2:** Effect of BCSE on CAT and GPx activities (U/mg protein) and GSH (mg/g protein) and MDA (nmol/mg protein) levels in the cerebrum of mercury-treated rats

Group	CAT	GPx	GSH	MDA
Normal	58.2±0.9	13.5±0.34	0.35±0.03	10.24±0.76
Extract	49.9±0.4	13.3±0.11	0.39±0.01	9.65±0.07
HgCl_2_	123.64±1.1*	10.6±0.37*	0.14±0.01*	24.42±0.44*
Extract I + HgCl_2_	59.27±0.1^#^	11.7±0.05	0.15±0.01	12.52±0.07^#^
Extract II + HgCl_2_	49.53±0.5^#^	13.9±0.08^#^	0.31±0.04^#&^	11.03±0.49^#^

Values are mean±SD (5 rats/group). *p<0.05: significant when compared to the normal control group in the same column. #p<0.05: significant when compared to the HgCl_2_ group in the same column. ^&^p<0.05: significant when compared to the Extract I + HgCl_2_ group in the same column.

In comparison, the 400 mg/kg dose significantly increased GSH than the GSH level obtained at 200 mg/kg dose. Additionally, BSCE significantly reduced MDA at 200 and 400 mg/kg compared to the HgCl_2_ group (p<0.05).

**Effect of BSCE on cerebellar oxidative stress indices**

Table 3 shows the effect of *B. coriacea *seed methanol extract (BCSE) on antioxidant enzymes CAT and GPx as well as GSH and MDA in the cerebellum of rats treated with Hg. Hg administration significantly increased CAT activity, while GPx activity was markedly reduced (p<0.05) compared to the normal control group. Also, Hg considerably reduced cerebellar level of GSH with prominent increases in MDA level compared to the normal control group (p<0.05). Conversely, administration of BCSE for 28 days moderated the CAT activity close to the normal control group rats, although significantly lower compared to the HgCl_2_ group. The GPx activity and GSH level were elevated markedly only at 400 mg/kg dose of BSCE for GPx compared to the HgCl_2_ group (p<0.05). Additionally, BSCE insignificantly reduced MDA compared to the HgCl_2_ group (p>0.05).

**Effect of BSCE on cerebral and cerebellar cholesterol, ADA, AchE and NO**

**Figure 1:**
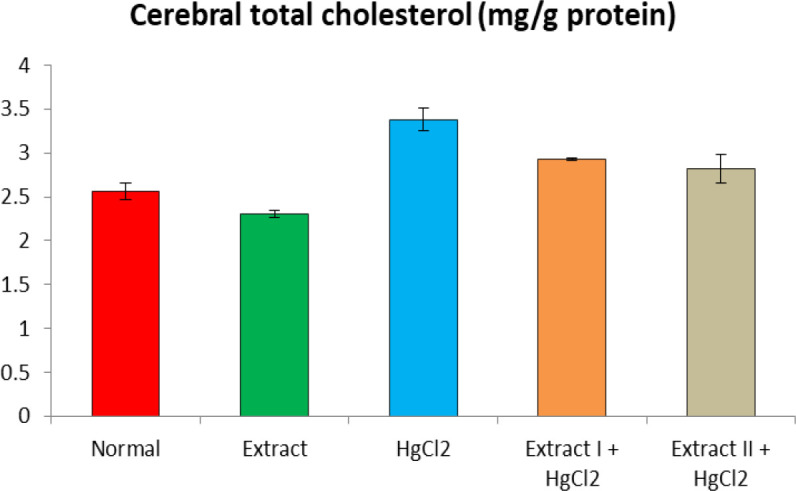
Effect of BCSE and HgCl_2_ on cerebral total cholesterol in HgCl_2_-treated rats. Values are mean±SD (5 rats/group)

**Figure 2:**
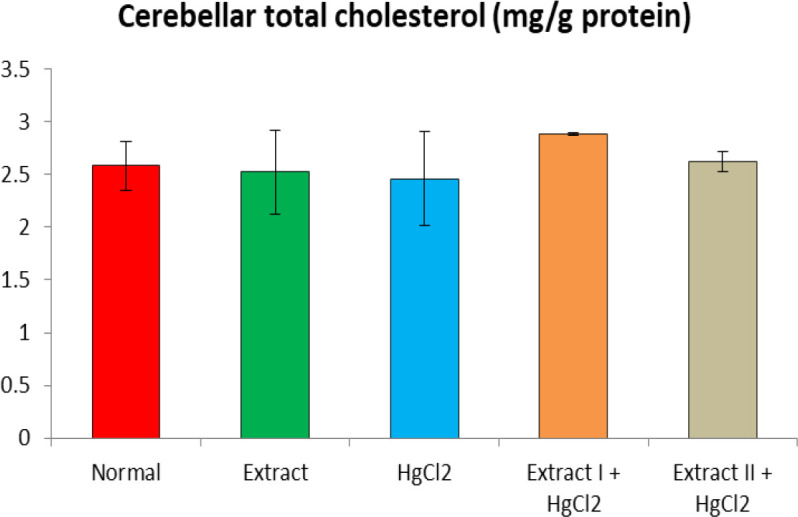
Effect of BCSE and HgCl_2_ on cerebellar total cholesterol in HgCl_2_-treated rats. Values are mean±SD (5 rats/group)

**Figure 3:**
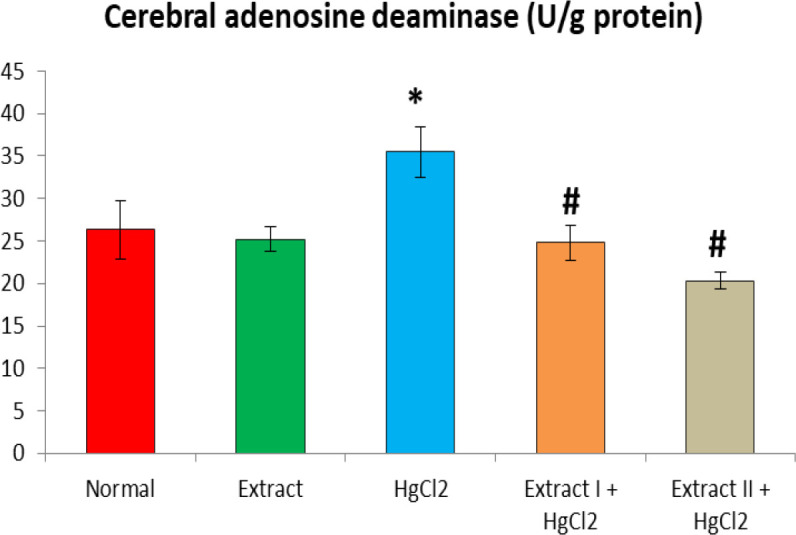
Effect of BCSE and HgCl_2_ on cerebral adenosine deaminase in HgCl_2_-treated rats. Values are mean±SD (5 rats/group). *p<0.05: significant when compared to normal control group. #p<0.05: significant when compared to HgCl_2_ group

**Figure 4:**
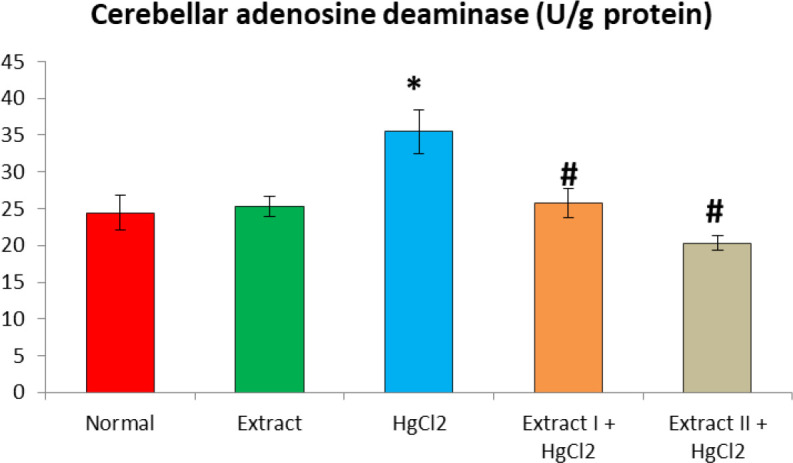
Effect of BCSE and HgCl_2_ on cerebellar adenosine deaminase in HgCl_2_-treated rats. Values are mean±SD (5 rats/group). *p<0.05: significant when compared to the normal control group. #p<0.05: significant when compared to the HgCl_2_ group

**Figure 5:**
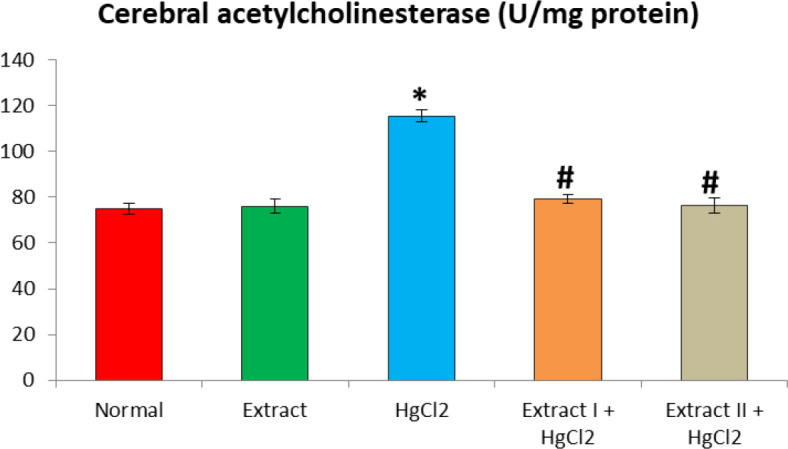
Effect of BCSE and HgCl_2_ on cerebral acetylcholinesterase in HgCl_2_-treated rats. Values are mean±SD (5 rats/group). *p<0.05: significant when compared to the normal control group. #p<0.05: significant when compared to the HgCl_2_ group

**Figure 6:**
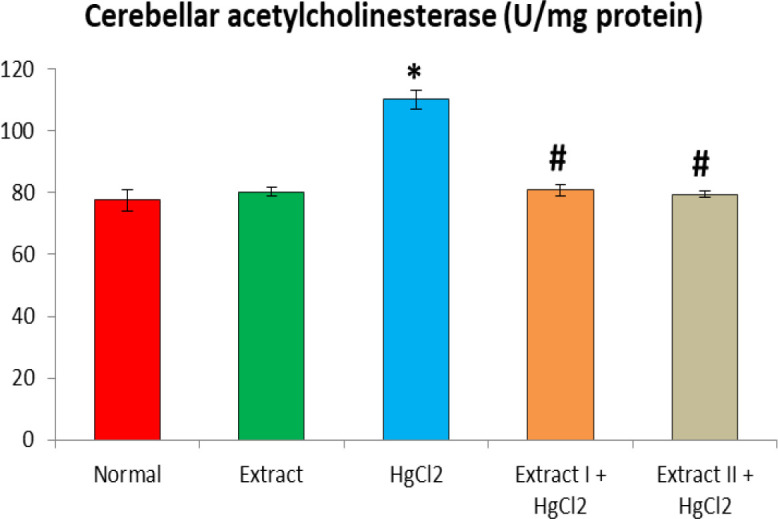
Effect of BCSE and HgCl_2_ on cerebellar acetylcholinesterase in the HgCl_2_-treated rats. Values are mean±SD (5 rats/group). *p<0.05: significant when compared to the normal control group. #p<0.05: significant when compared to the HgCl_2_ group

**Figure 7:**
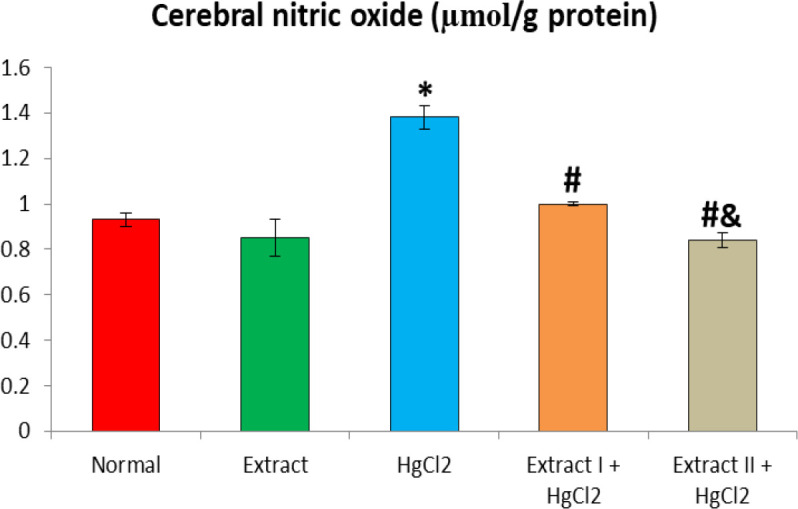
Effect of BCSE and HgCl_2_ on cerebral nitric oxide in the HgCl_2_-treated rats. Values are mean±SD (5 rats/group). *p<0.05: significant when compared to the normal control group. #p<0.05: significant when compared to the HgCl_2_ group. &p<0.05: significant when compared to the Extract I + HgCl_2_ group

**Figure 8:**
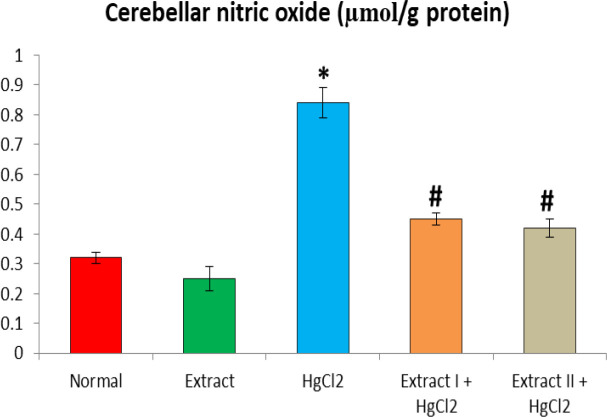
Effect of BCSE and HgCl_2_ on cerebellar nitric oxide in the HgCl_2_-treated rats. Values are mean±SD (5 rats/group). *p<0.05: significant when compared to the normal control group. #p<0.05: significant when compared to the HgCl_2_ group

Figure 1 to 8 depict the effect of BCSE on cerebral and cerebellar levels of cholesterol, ADA, AchE and NO in HgCl_2_-treated rats. Hg induced insignificant (p>0.05) effects on the levels of total cholesterol in the brain parts. A similar effect was observed after the administration of BCSE which demonstrated insignificant effects on total cholesterol (p>0.05). However, Hg exposure for the 14 days (last two weeks) induced significant increases in the cerebral and cerebellar NO levels and ADA and AchE activities compared to the normal control group (p<0.05). Interestingly, BCSE sub-acute prophylactic administration attenuated the increases shown by prominent decreases in NO level and ADA and AchE activities compared to the HgCl_2_ group. Notably, the effect of BCSE on cerebral NO level was dose-dependent.

**Effect of BSCE on brain histopathology**

The photomicrograph of the cerebrum and cerebellum showed normal histology. Normal neuronal cell bodies (N), vascularized glial cells (G), capillaries (C) and neutrophil (Np) were indicated in the cerebrum, while molecular layer (M), the Purkinge cells (black arrow) and the granular cells (white arrow) of the granular layer (G) were shown in cerebellum. In the Hg-treated group, neuronal cell bodies (N) appeared shrunken, with pyknotic nuclei and deeply basophilic and shrunken cytoplasm in the cerebrum and cerebellum. The cerebellum of the Hg-treated rats showed degeneration and necrosis of the purkinje cells (black arrow) and granular cells (white arrow) of the granular layer. In the Extract + HgCl_2_ group, comparatively, the brain histopathological alterations were alleviated by the BCSE treatment.

**Table 3:** Effect of BCSE on CAT and GPx activities (U/mg protein) and GSH (mg/g protein) and MDA (nmol/mg protein) levels in the cerebellum of mercury-treated rats

Group	CAT	GPx	GSH	MDA
Normal	60.19±0.29	13.5±0.33	0.32±0.03	12.22±0.50
Extract	77.84±0.33^&^	13.9±0.12	0.41±0.01^&^	8.47±0.08^&^
HgCl_2_	106.1±3.22*	12.1±0 .62*	0.26±0.01*	23.96±0.54*
Extract I + HgCl_2_	99.21±0.01	13.7±0.36	0.32±0.01^#^	20.68±0.05
Extract II + HgCl_2_	80.7±0.04^#^	14.0±0.41^#^	0.35±0.07^#^	20.56±0.06

Values are mean±SD (5 rats/group). ^&^p<0.05: significant when compared to the normal control group in the same column. *p<0.05: significant when compared to the normal control group in the same column. #p<0.05: significant when compared to the HgCl_2_ group in the same column.

**Figure 9:**
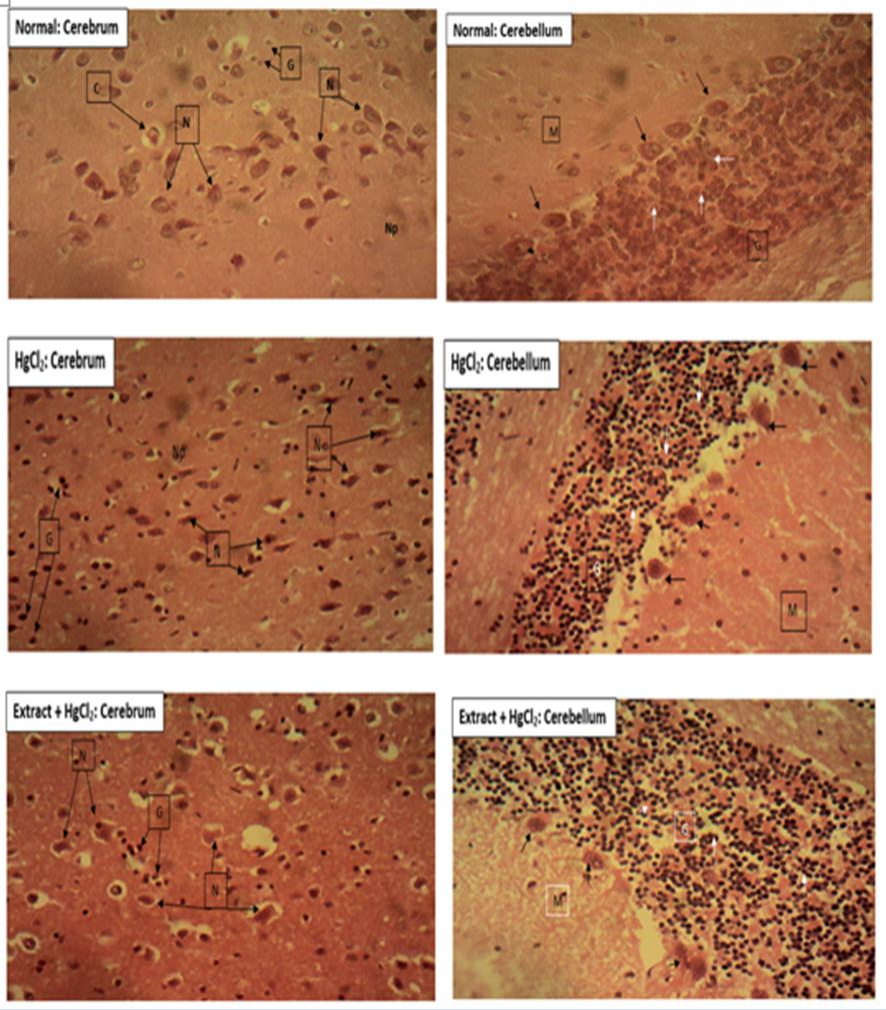
Photomicrographs of histopathological examination of cerebrum and cerebellum of rats treated with HgCl_2_. Normal: normal histology; HgCl_2_: disruption of the normal cerebral and cerebellar architecture by HgCl_2_ administration was observed as shown by arrows. Treatment with *Buccholzia coriacea* seed methanol extract (BCSE) showed protective changes in the histological structures to almost similar to the control group

## Discussion

In the present study, it was observed that Hg exerts depression of GPx activity and GSH level in the cerebrum and cerebellum of rats. Consequently, lipid peroxidation was triggered as indicated by the considerably increased cerebral and cerebellar MDA level, whereas, the CAT activity increased markedly. Abundant evidence has confirmed that Hg induces oxidative stress in the brain of rats via reducing the activities of CAT and GPx, level of GSH, and consequent elevation of MDA (Ansar, 2015 ▶; Moneim, 2015 ▶; Lucena et al., 2007 ▶). In this regard, our findings here are in consonance with previous reports (Salman et al., 2016 ▶; Ansar, 2015 ▶; Moneim, 2015 ▶; Lucena et al., 2007 ▶). The mechanism of Hg inhibition of antioxidant enzymes and molecules is recognized to be associated with Hg ability to react with and deplete sulfhydryl (-SH) groups present in GPx and GSH (Adedara et al., 2019 ▶; Mesquita et al., 2016 ▶; Moneim, 2015 ▶; Sutton and Tchounwou 2006 ▶). The Hg-SH complex formed inactivates sulfhydryl group-containing enzymes and GSH. The literature supports that reduction in GSH, a master reductant mediator of redox homeostasis, may induce redox imbalance significantly (Aoyama and Nakaki, 2013 ▶). This concomitantly triggers an oxidative stress in brain cells leading to oxidative neurotoxicity underlying biochemical and histopathological observations of the current study. Glutathione peroxidase (GPx) utilizes GSH to reduce oxidized lipids and protein targets of ROS to avert the course of lipid peroxidation (Famurewa et al., 2017 ▶). Due to depressed activity of GPx and GSH level, peroxidation of lipids was aggravated evident by increased MDA level in the cerebrum and cerebellum. However, it is noteworthy that an increased CAT activity was found in our study. Under acute exposure to an oxidant, there could be a surge in antioxidant enzyme activity before the eventual decline in chronic oxidant exposure (Vardi et al., 2008 ▶). Some authors found that oxidative stress milieu induces prominent increases in superoxide dismutase and CAT activities in the cerebellum and small intestine of rats (Uzar et al., 2006a ▶; Miyazono et al., 2004 ▶). Related report further shows that methyl mercury increased cerebellar glutathione reductase activity (Lucena et al., 2007 ▶). According to Vardi et al. (2010) ▶, mild to moderate cellular oxidative stress may increase the protein expression and activity of antioxidant enzymes as a compensatory mechanism of protection against possible damage. However, very high levels of toxicants may down-regulate enzyme activities due to damages induced in the molecular machinery required to induce these enzymes (Vardi et al., 2008 ▶). Interestingly, the prophylactic treatment with the *B. coriacea* seeds methanol extract (BCSE) attenuated and restored the altered oxidative stress indices. This was demonstrated by increased activity of GPx and GSH level, while MDA level decreased significantly in the cerebrum and cerebellum, indicating reduced lipid peroxidation. The antioxidant activity of BCSE has been reported (Adisa et al., 2011 ▶). It contains alkaloids, anthraquinones, saponins, tanins, cardiac glycosides and flavonoids that may act directly against ROS or via improving and preserving the antioxidant enzyme activity comparable to the control groups in this study (Eze et al., 2017 ▶; Adisa et al., 2011 ▶). The reduction of MDA level and elevation of GPx and GSH level by BCSE suggest that *B. coriacea* seeds possess an antioxidant property contributed by the phytochemicals previously reported in this study.

Toxic metal exposure has been reported to invoke lipid dysfunction in humans and animals (Cho, 2017 ▶; Pandya et al., 2004 ▶). Currently, evaluation of the effect of Hg exposure on brain cholesterol is scarce. In our study, sub-acute Hg exposure demonstrated non-significant total cholesterol changes in the cerebrum and cerebellum in comparison to the normal control. In previous studies, long-term exposure to Hg via heavy consumption of fish was associated with increased blood Hg level and hypercholesterolemia (Cho, 2017 ▶). In rats, aluminium exposure for 5 months was implicated to induce impaired levels of cholesterol in the posterior brain (Belaïd-Nouira et al., 2012 ▶). Considering the chronic exposure considered in the earlier studies, the sub-acute (28 days) Hg exposure in our study may be the cause for the non-significant effect of Hg on total cholesterol. 

Cholinergic system plays a critical role in neurotransmission, learning and memory functions. Toxic metals are known to disrupt cholinergic mechanism through interactions with AchE and neurotransmitters (Akinyemi et al., 2015 ▶). In this study, our analyses revealed that Hg increased AchE activity in the cerebrum and cerebellum. AchE hydrolyzes acetylcholine in the brain, and has been implicated in metal toxicity (Ansar, 2015 ▶). Our result is consistent with the previous reports of increased AchE activity in the brain after exposures to lead and cadmium (Akinyemi et al., 2015 ▶; Gill et al., 1991 ▶). Studies suggest that the toxic metals might interact with AchE and/or AchE receptors thus increasing its activity to hydrolyze Ach (Romani et al., 2003 ▶). By implication, the activation of AchE suggests that Hg exposure may induce cognitive or cholinergic deficit (Ishola et al., 2017 ▶). Furthermore, cerebral and cerebellar ADA and NO levels were adversely significantly increased by Hg compared to the normal control. ADA acts to deaminate adenosine to inosine; however, the action of xanthine oxidase on inosine to produce uric acid was suggested an important source of superoxide anion (Fadillioglu et al., 2003 ▶). NO is synthesized by inducible nitric oxide synthase (iNOS); it reacts with superoxide anion to peroxynitrite radical (ONOO^-^), an aggressive and potent oxidant that causes oxidation or nitrosylation of sulfydryl group, DNA and brain damage (Famurewa et al., 2019 ▶; Abdel-Salam et al., 2016 ▶; Uzar et al., 2006b ▶). It is evident thus that the Hg-induced increases in ADA and NO may exacerbate oxidative damage to the brain regions explored in this study. The increased NO level in the current study after Hg exposure may be due to the activation of iNOS and nuclear factor-kappa B (NF-ĸB) signalling (Moneim, 2015 ▶). Literature indicates that increased NO signalling is associated with activation of NF-ĸB/iNOS neuroinflammatory signalling which could culminate into cell death (Mahmud et al., 2017 ▶; Hegazy et al., 2016 ▶). Our results hereby agree with previous findings from animal models (Hegazy et al., 2016 ▶; Moneim, 2015 ▶; Uzar et al., 2006b ▶). In contrast, BCSE decreased AchE and ADA activities and NO level in the brain regions. Previous studies indicated that antioxidant compounds or extract from medicinal plants could reverse or modulate levels of NO, ADA and AchE activities in the brain (Uzar et al., 2006b ▶). Although, phytochemical profiling was not explored here, earlier studies have indicated that BCSE contains flavonoids, glycosides and saponins known to modulate AchE and ADA activities and NO level (Adisa et al., 2011 ▶; Ajaiyeoba et al., 2001 ▶). The BCSE doses (200 and 400 mg/kg) revealed a dose-dependent effect on Hg-induced oxidative neurotoxicity although insignificantly when compared. However, the the higher dose (400 mg/kg) of BCSE significantly reduced cerebral NO level compared to the lower dose (200 mg/kg) of BCSE. 

In conclusion, our study reinforced the previous studies on toxic capacity of Hg to induce neurotoxicity. The neuroprotective effect of BCSE was demonstrated, for the first time, against Hg-induced oxidative stress-mediated neurotoxicity. The protective mechanism evidently involves decreased oxidative stress and suppression of AchE, ADA and NO generation in the cerebrum and cerebellum of rats. This beneficial health effect may involve the bioactive action of antioxidant phytochemicals resident in BCSE. BCSE might thus find a role in mitigating brain damage following exposure to Hg exposure. 

## Conflicts of interest

The authors have declared that there is no conflict of interest.

## References

[B1] Abdel-Salam OM, Youness ER, Mohammed NA, Yassen NN, Khadrawy YA, El-Toukhy SE, Sleem AA (2016). Novel neuroprotective and hepatoprotective effects of citric acid in acute malathion intoxication. Asian Pac J Trop Med.

[B2] Adedara IA, Fasina OB, Ayeni MF, Ajayi OM, Farombi EO (2019). Protocatechuic acid ameliorates neurobehavioral deficits via suppression of oxidative damage, inflammation, caspase-3 and acetylcholinesterase activities in diabetic rats. Food Chem Toxicol.

[B3] Adisa RA, Choudhary MI, Olorunsogo OO (2011). Hypoglycemic activity of Buchholzia coriacea (Capparaceae) seeds in streptozotocin-induced diabetic rats and mice. Exp Toxicol Pathol.

[B4] Olaiya CO, Omolekan TO (2013). Antihypercholesterolemic activity of ethanolic extract of Buchholzia coriacea in rats. Afr Health Sci.

[B5] Aebi H, H. U. Bergmeyer (1983). Catalase. Methods in enzymatic analysis.

[B6] Ajaiyeoba EO, Onocha PA, Olanrewaju OT (2001). In vitro anti-helminthic properties of Buchholzia coriacea and Gynandropsis gynandra. Pharm Biol.

[B7] Akinyemi AJ, Oboh G, Ademiluyi AO (2015). Local salt substitutes “Obu-otoyo” activate acetylcholinesterase and butyrylcholinesterase and induce lipid peroxidation in rat brain. Interdis Toxicol.

[B8] Ansar S (2015). Pretreatment with diallylsulphide modulates mercury-induced neurotoxicity in male rats. Acta Biochim Pol.

[B9] Aoyama K, Nakaki T (2013). Impaired glutathione synthesis in neurodegeneration. Int J Mol Sci.

[B10] Bainy AD, de Medeiros MDH, Di Mascio P, de Almeida EA (2006). In vivo effects of metals on the acetylcholinesterase activity of the Perna perna mussel’s digestive gland. Biotemas.

[B11] Bakulski KM, Lee H, Feinberg JI, Wells EM, Brown S, Herbstman JB, Witter FR, Halden RU, Caldwell K, Mortensen ME, Jaffe AE, Moye J, Caulfield LE, Pan Y, Goldman LR, Feinberg AP, Fallin MD (2015). Prenatal mercury concentration is associated with changes in DNA methylation at TCEANC2 in newborns. Int J Epidemiol.

[B12] Belaïd-Nouira Y, Bakhta H, Bouaziz M, Flehi-Slim I, Haouas Z, Cheikh HB (2012). Study of lipid profile and parieto-temporal lipid peroxidation in AlCl3 mediated neurotoxicity: Modulatory effect of fenugreek seeds. Lipids Health Dis.

[B13] Cardenas A, Rifas-Shiman SL, Godderis L, Duca R, Navas-Acien A, Litonjua AA, DeMeo DL, Brennan KJ, Amarasiriwardena CJ, Hivert M-F, Gillman MW, Oken E, Baccarelli AA (2017). Prenatal exposure to mercury: associations with global DNA methylation and hydroxymethylation in cord blood and in childhood. Environ Health Perspec.

[B14] Castoldi AF, Coccini T, Manzo L (2003). Neurotoxic and molecular effects of methylmercury in humans. Rev Environ Health.

[B15] Cho YM (2017). Fish consumption, mercury exposure, and the risk of cholesterol profiles: findings from the Korea National Health and Nutrition Examination Survey 2010-2011. Environ Health Toxicol.

[B16] Ellman GL, Courtney KD, Andres VJ, Feather‐Stone RM (1961). A new and rapid colorimetric determination of acetylcholinesterase activity. Biochem Pharmacol.

[B17] Eze JI, Ekelozie CF, Nweze NE (2017). Immunomodulatory activity of Buchholzia coriacea seed methanol extract on Trypanosoma brucei brucei infected mice. Pharm Biol.

[B18] Fadillioglu E, Yilmaz HR, Erdogan H, Sogut S (2003). The activities of tissue xanthine oxidase and adenosine deaminase and the levels of hydroxyproline and nitric oxide in rat hearts subjected to doxorubic: protective effect of erdosteine. Toxicol.

[B19] Famurewa AC, Aja PM, Nwankwo OE, Awoke JN, Maduagwuna EK, Aloke C (2019). Moringa oleifera seed oil or virgin coconut oil supplementation abrogates cerebral neurotoxicity induced by antineoplastic agent methotrexate by suppression of oxidative stress and neuroinflammation in rats. J Food Biochem.

[B20] Famurewa AC, Ufebe OG, Egedigwe CA, Nwankwo OE, Obaje GS (2017). Virgin coconut oil supplementation attenuates acute chemotherapy hepatotoxicity induced by anticancer drug methotrexate via inhibition of oxidative stress in rats. Biomed Pharmacother.

[B21] Flohe L, Gunzler W, S. P. Colowick (1984). Assays of glutathione peroxidase. Methods enzymology.

[B22] Gill TS, Teware H, Pande J (1991). In vivo and in vitro eff ects of cadmium on selected enzymes in diff erent organs of the fish Barbus conchonius Ham. (Rosy barb). Comp Biochem Physiol Part C.

[B23] Giusti G, Bergmeyer, M (1974). Adenosine deaminase. Methods of Enzymatic Analysis.

[B24] Goudarzi M, Kalantar M, Kalantar H (2017). The hepatoprotective effect of gallic acid on mercuric chloride-induced liver damage in rats. Jundishapur J Natural Pharm Prod.

[B25] Hegazy HG, Ali EH, Sabry HA (2016). The neuroprotective action of naringenin on oseltamivir (Tamiflu) treated male rats. J Basic Appl Zool.

[B26] Ishola IO, Adamson FM, Adeyemi OO (2017). Ameliorative effect of kolaviron, a biflavonoid complex from Garcinia kola seeds against scopolamine-induced memory impairment in rats: role of antioxidant defense system. Metab Brain Dis.

[B27] Jollow DJ, Michell JR, Zampaglione N, Gillete J (1974). Bromobenzene‐induced liver necrosis: Protective role of glutathione and evidence for 3, 4‐bromobenzene oxide as the hepatotoxic metabolite. Pharmacol.

[B28] Lakshmana MK, Desiraju T, Raju TR (1993). Mercuric chloride-induced alterations of levels of noradrenaline, dopamine, serotonin and acetylcholine esterase activity in different regions of rat brain during postnatal development. Arch Toxicol.

[B29] Lucena GM, Franco JL, Ribas CM, Azevedo MS, Meotti FC, Gadotti VM, Dafre AL, Santos AR, Farina M (2007). Cipura paludosa extract prevents methyl mercury-induced neurotoxicity in mice. Basic Clin Pharmacol Toxicol.

[B30] Mahmud HA, Seo H, Kim S, Islam MI, Nam K-W, Cho H-D, Song H-Y (2017). Thymoquinone (TQ) inhibits the replication of intracellular Mycobacterium tuberculosis in macrophages and modulates nitric oxide production. BMC Complement Altern Med.

[B31] Mesquita M, Pedroso TF, Oliveira CS, Oliveira VA, Francisco do Santos R, Bizzi CA, Pereira ME (2016). Effects of zinc against mercury toxicity in female rats 12 and 48 hours after HgCl2 exposure. EXCLI J.

[B32] Mieiro CL, Pereira ME, Duarte AC, Pacheco M (2011). Brain as a critical target of mercury in environmentally exposed fish (Dicentrarchus labrax)--bioaccumulation and oxidative stress profiles. Aquatic Toxicol.

[B33] Miyazono Y, Gao F, Horie T (2004). Oxidative stress contributes to methotrexate- induced small intestinal toxicity in rats. Scand J Gastroenterol.

[B34] Moneim AE (2015). The neuroprotective effect of berberine in mercury-induced neurotoxicity in rats. Metab Brain Dis.

[B35] Ohkawa H, Ohishi N, Yagi K (1979). Assay for lipid peroxides in animal tissues by thiobarbituric acid reaction. Annals Biochem.

[B36] Owoeye O, Gabriel MO (2016). Protective effects of aqueous extract of Telfairia occidentalis on mercury-induced histological and oxidative changes in the rat hippocampus and cerebellum. Afr J Biomed Res.

[B37] Pandya JD, Dave KR, Katyare SS (2004). Effect of long-term aluminum feeding on lipid/phospholipid profiles of rat brain myelin. Lipids Health Dis.

[B38] Rao MV, Purohit AR (2011). Neuroprotection by melatonin on mercury induced toxicity in the rat brain. Pharmacol Pharm.

[B39] Romani R, Antognelli C, Baldracchini F, De Santis A, Isani G, Giovannini E (2003). Increased acetylcholinesterase activities in specimens of Sparus auratus exposed to sub lethal copper concentrations. Chem Biol Interact.

[B40] Salman MM, Kotb AM, Haridy MA, Hammad S (2016). Hepato- and nephroprotective effects of bradykinin potentiating factor from scorpion (Buthus occitanus) venom on mercuric chloride-treated rats. EXCLI J.

[B41] Sreejayam N, Rao MN (1997). Nitric oxide scavenging activity by curcummoids. J Pharm Pharmacol.

[B42] Sutton DJ, Tchounwou PB (2006). Mercury-induced externalization of phosphatidylserine and caspase 3 activation in human liver carcinoma (HepG2) cells. Int J Environ Res Public Health.

[B43] Thiagarajan K, Gamit N, Mandal S, Ayyathan DM, Chandrasekaran R (2018). Amelioration of methylmercury induced neural damage by essential oil of Selinum vaginatum (Edgew) C. B. Clarke. Pak J Pharm Sci.

[B44] Uzar E, Koyuncuoglu HR, Uz E, Yilmaz AR, Kutluhan S, Kilbas S, Gultekin F (2006a). The activities of antioxidant enzymes and the level malondialdehyde in cerebellum of rats subjected to methotrexate: protective effect of caffeic acid phenetyl ester. Mol Cell Biochem.

[B45] Uzar E, Sahin O, Koyuncuoglu HR, Uz E, Bas O, Kilbas S, Yilmaz HR, Yurekli VA, Kucuker H, Songur A (2006b). The activity of adenosine deaminase and the level of nitric oxide in spinal cord of methotrexate administered rats: Protective effect of caffeic acid phenethyl ester. Toxicol.

[B46] Vardi N, Parlakpinar H, Ozturk F, Ates B, Gul M, Cetin A, Erdogan A, Otlu A (2008). Potent protective effect of apricot and β-carotene on methotrexate-induced intestinal oxidative damage in rats. Food Chem Toxicol.

[B47] Vardi N, Parlakpinar H, Cetin A, Erdogan A, Ozturk IC (2010). Protective effect of beta-carotene on methotrexate-induced oxidative liver damage. Toxicol Pathol.

